# Do preeclampsia symptoms resolve after intrauterine death of a fetus?

**DOI:** 10.4274/tjod.84770

**Published:** 2016-06-15

**Authors:** Serdar Balcı, Taylan Bodur, Yusuf Aytaç Tohma, Recep Emre Okyay, Bahadır Saatli, Sabahattin Altunyurt

**Affiliations:** 1 Başkent University Faculty of Medicine, Department of Obstetrics and Gynecology, Ankara, Turkey; 2 Muğla Yücelen Hospital, Clinic of Obstetrics and Gynecology, Muğla, Turkey; 3 Dokuz Eylül University Faculty of Medicine, Department of Obstetrics and Gynecology, İzmir, Turkey

**Keywords:** preeclampsia, twin pregnancy, growth restriction

## Abstract

We present two cases of twin pregnancies without resolution of preeclamptic symptoms after intrauterine death of one twin.

**Case 1:**

A nulliparous woman aged 37 years was referred at 26 weeks of gestation because of arterial hypertension, edema, and growth restriction in one twin. Three weeks later the restricted twin died. During the following three weeks, ultrasound examinations showed a reduced growth velocity of the surviving fetus and reversed umbilical flow. At the end of the 34^th^ week of gestation, cesarean section was performed and a healthy female infant was delivered.

**Case 2:**

A nulliparous woman aged 33 years with a 27-week twin pregnancy was referred because of arterial hypertension and discordant growth. The restricted twin died at 31 weeks of gestation. Following the death, within two weeks the growth of the co-twin started to slow down and reversed end diastolic flow presented. At the end of the 33^rd^ week of gestation, cesarean section was performed and a healthy female infant was delivered.

The interesting point of these cases was the secondary effects on the co-twins. During the time after intrauterine deaths of one twin, the surviving fetuses started to show a reduced growth velocity and reversed umbilical flow and mothers had increased blood pressure and proteinuria again. We think that both cases are evidence of late on-set systemic maternal effects (such as systemic maternal endothelial activation and/or systemic maternal inflammatory response) depends on preeclampsia.

## INTRODUCTION

Preeclampsia originates from the placenta, and its progressive clinical course is only treated by delivery of the placenta^(1)^. The incidence of disease is 3-5% and it is known as a major cause of maternal and perinatal mortality^(2)^. Although the etiology and pathogenesis remain to be clarified, termination of pregnancy or delivery of the placenta eradicates the disease; therefore, the placenta is undoubtedly related to preeclampsia^(3)^. Twin gestations occur in 3.2% of pregnancies and are associated with increased risks of gestational diabetes mellitus, preterm delivery, intrauterine growth restriction, hypertension and hemorrahage^(4)^. Additionally, the risk of preeclampsia in twin pregnancies increases more than twice compared with singleton pregnancies^(4)^. We present two cases of twin pregnancies without resolution of preeclamptic symptoms after the intrauterine death of one twin.

## CASE REPORTS

### Case 1

A nulliparous woman aged 37 years was referred at 26 weeks of gestation because of arterial hypertension, edema, and growth restriction in one twin. On admission her blood pressure was 160/100 mmHg. In our centre, ultrasound examination confirmed growth restriction (weight estimation: 389 g) and reversed umbilical flow with cerebral redistribution in one twin, with a normal co-twin (estimated weight: 855 g). This was a dichorionic, diamniotic twin pregnancy. The quantitative analysis of proteinuria in a 24-hour urine sample taken after admission showed 2.708 g/day. To avoid prematurity, expectant management was planned. The patient received alpha-methyldopa in a dosage of 1000 mg daily for blood pressure control and magnesium sulfate at a daily dosage of 1500 mg to prevent convulsions. Two days later, because of intolerable nausea and vomiting, we had to stop the magnesium sulfate but we kept going on alpha-methyldopa. During the following three weeks, the blood pressure was under partial control, ultrasound examinations showed no evidence of growth of the restricted twin and confirmed severe doppler abnormalities; the co-twin’s assessment was reassuring. Some days later restricted twin died but doppler flows of the surviving twin were normal. Accordingly, we decided to prolong the pregnancy under close observation. Significant proteinuria occured again at 31 weeks of gestation, accompanied by a rise in blood pressure. During the following three weeks, ultrasound examinations showed a reduced growth velocity of the surviving fetus and reversed umbilical flow. At the end of the 34th week of gestation, cesarean section was performed and a healthy female infant weighing 1670 g was delivered, followed by a macerated female fetus of nearly 200 g. The mother recoverd quickly and her blood pressure was normal on the third day postpartum.

### Case 2

A nulliparous woman aged 33 years with a 27-week twin pregnancy was referred because of arterial hypertension and discordant growth. On admission her blood pressure was 170/105 mmHg. Our ultrasound check showed growth restriction (weight estimation: 698 g) and reversed umbilical flow in one twin, with a normal co-twin (estimated weight: 1.060 g). The examination also revealed a dichorionic, diamniotic twin pregnancy. In a 24-hour urine sample showed proteinuria (4.850 g/day). Expectant management was planned to prevent prematurity. The patient received alpha-methyldopa in a daily dosage of 1000 mg for blood pressure control and magnesium sulfate at a dosage of 1500 mg daily to prevent convulsions. During the following two weeks, beside the partial blood pressure control, ultrasound examinations revealed no growth in the restricted twin and abnormal umblical artery flow; the co-twin’s assessment was good. At 31 weeks of gestation, the restricted twin died and a significant proteinuria (6.650 g/day) reappeared but the co-twins doppler flows were normal. Following the death, growth of co-twin started to draw back within two weeks and reversed end diastolic flow presented. At the end of the 33rd week of gestation, cesarean section was performed and a healthy female infant weighing 1.370 g was delivered, followed by a macerated female fetus of almost 800 g. We discharged the mother from hospital after her blood pressure normalized on the fourth day postpartum.

## DISCUSSION

It is certain that the placenta takes a central role in the pathogenesis of preeclampsia. The importance of poor placentation as a feature of the disorder is well documented^(5)^. However, we still do not know the reasons of poor placentation.

In these cases there are two separate placentas. One of them is preeclamptic, the pathologic report documented low weights with fibrin and thrombosis inside the vessels, and the others were normal. We still do not know why one of the twin placentas has abnormal development when both have the same maternal factors such as immunity or blood pressure. One explanation is local factors such as fetus or placental localization, which could possibly affect placental growth.

Another interesting point of these cases was the secondary effects on the co-twins. After intrauterine deaths of one twin, the surviving fetuses started to show a reduced growth velocity and reversed umblical flow and the mothers had increased blood pressure and proteinuria again. We think that this is evidence of late on-set systemic maternal effects such as systemic maternal endothelial activation and/or systemic maternal inflammatory response) depends on preeclampsia.

Usually preeclampsia progresses without any recovery until birth. Few cases of resolution of preeclampsia after spontaneous intrauterine death of one twin or selective termination of a dichorionic pregnancy have been reported^(1,6,7)^. Narasimhulu et al.^(8)^ reported resolution of superimposed preeclampsia in a surviving fetus after the intrauterine demise of its co-twin and suggested that placental involution after fetal demise was the key to resolution of preeclampsia (resolution period take 1 to 3 weeks). However, according to our cases, even if the fetus dies, preeclampsia could not be curable unless placental seperation occurred. The greatest disparity of our cases compared with those in the literature was the amount of proteinuria, nearly 5 g/day in 24-hour urine sample. Consequently, severe preeclampsia may differ from mild preeclampsia, without any resolution or resolution in longer time (more than 3 weeks). In addition, a retrospective analysis of outcomes in pregnant women who had expectant managemant with severe preeclampsia at less than 27 weeks’ gestation revealed that overall perinatal survival was 57% (9% of them were multifetal gestations)^(9)^. As a result, resolution of preeclampsia in dichorionic twin pregnancies following intrauterine death of restricted fetus is still uncertain, especially in severe preeclampsia.

To our knowledge, these are the first cases to describe the return of preeclamptic symptoms in twins after the intrauterine death of one twin in the literature. Further studies are needed for the comprehension of preeclampsia and recognition of placental factors responsible for the persistence of the disease.
